# Aberrant methylation of *Pax3* gene and neural tube defects in association with exposure to polycyclic aromatic hydrocarbons

**DOI:** 10.1186/s13148-019-0611-7

**Published:** 2019-01-21

**Authors:** Shanshan Lin, Aiguo Ren, Linlin Wang, Chloe Santos, Yun Huang, Lei Jin, Zhiwen Li, Nicholas D. E. Greene

**Affiliations:** 1 Institute of Reproductive and Child Health, National Health Commission Key Laboratory of Reproductive Health, and Department of Epidemiology and Biostatistics, School of Public Health, Peking University Health Centre, Peking University, Beijing, 100191 China; 2 Division of Birth Cohort Study, and Department of Neonatal Surgery, Guangzhou Women and Children’s Medical Centre, Guangzhou Medical University, Guangzhou, China; 3 Developmental Biology and Cancer Programme, UCL Great Ormond Street Institute of Child Health, University College London, London, WC1N 1EH UK

**Keywords:** Neural tube defects, *Pax3* gene, Methylation, Benzo[a]pyrene, Polycyclic aromatic hydrocarbon

## Abstract

**Background:**

Neural tube defects (NTDs) are common and severe congenital malformations. *Pax3* is an essential gene for neural tube closure in mice but it is unknown whether altered expression or methylation of *PAX3* contributes to human NTDs. We examined the potential role of hypermethylation of *Pax3* in the development of NTDs by analyzing human NTD cases and a mouse model in which NTDs were induced by benzo[a]pyrene (BaP), a widely studied polycyclic aromatic hydrocarbon (PAH).

**Methods:**

We extracted methylation information of *PAX3* in neural tissues from array data of ten NTD cases and eight non-malformed controls. A validation study was then performed in a larger independent population comprising 73 NTD cases and 29 controls. Finally, we examined methylation patterns and expression of *Pax3* in neural tissues from mouse embryos of dams exposed to BaP or BaP and vitamin E.

**Results:**

Seven CpG sites in *PAX3* were hypermethylated in NTD fetuses as compared to controls in the array data. In the validation phase, significantly higher methylation levels in the body region of *PAX3* were observed in NTD cases than in controls (*P* = 0.003). And mean methylation intensity in the body region of *PAX3* in fetal neural tissues was positively correlated with median concentrations of PAH in maternal serum. In the mouse model, BaP-induced NTDs were associated with hypermethylation of specific CpG sites within both the promoter and body region of *Pax3*. Supplementation with vitamin E via chow decreased the rate of NTDs, partly recovered the repressed total antioxidant capacity in mouse embryos exposed to BaP, and this was accompanied by the normalization of *Pax3* methylation level and gene expression.

**Conclusion:**

Hypermethylation of *Pax3* may play a role in the development of NTDs; DNA methylation aberration may be caused by exposure to BaP, with possible involvement of oxidative stress.

**Electronic supplementary material:**

The online version of this article (10.1186/s13148-019-0611-7) contains supplementary material, which is available to authorized users.

## Background

Neural tube defects (NTDs) arise from a failed or disordered closure of the neural tube during embryogenesis. The occurrence of NTDs is around 0.5–2/1000 pregnancies worldwide [1]. Fetuses affected with an NTD are often stillborn (e.g., anencepahlics), and most surviving infants suffer from life-long disabilities. The etiology of NTDs is complex, involving both genetic and non-genetic factors [2]. Over 300 genes have been identified to be involved in the regulation of neural tube closure in mouse NTD mutants [3, 4]; however, only a few of these genes have successfully been validated in human NTDs. In addition to potential additive effects of multiple risk alleles, increasing attention has also focused on the potential for epigenetic alterations to contribute to the occurrence of NTDs by mediating the interplay of fetal genetics and environmental factors [5].

Epigenetic modification can cause changes in gene expression that are not directly related to the DNA sequence itself, of which DNA methylation is one of the best understood epigenetic mechanisms [6]. During early development, a tight regulation of genome-wide erasure of epigenetic footprints with resetting of the methylation signature takes place, making developing fetuses particularly susceptible to epigenetic dysregulation as a consequence of environmental exposure [7, 8]. Abnormal genome-wide methylation during embryogenesis has been linked to developmental abnormalities at birth, including NTDs. Recent studies have demonstrated that global DNA hypomethylation, evaluated using LINE-1 methylation as an indicator in human fetuses, was associated with an increased risk for NTDs [9]. Aberrations of methylation at specific genes are reported to be involved in NTDs, including imprinted genes [10, 11], DNA repair genes [12], planar-cell polarity genes [13, 14], and *HOX* genes [15].

Pax3 is a paired-homeodomain-containing transcription factor essential for promoting neural crest induction, maintenance, migration, and differentiation [16]. Previous studies in mice have shown that Pax3 function is required for neural tube closure. Several alleles of *Pax3* cause NTDs in mice and homozygous Splotch (*Sp*^*2H*^) embryos develop NTDs with 100% penetrance [17]. However, the role of *PAX3* in human NTDs remains unclear. Recessive and dominant mutations in *PAX3* in humans are known to cause Waardenburg syndrome, an autosomal dominant condition that affects neural crest-derived structures and also includes spina bifida as part of its phenotypic spectrum [18]. A 5-bp deletion in exon 5 of the *PAX3* gene was reported in a patient with spina bifida [19]. In contrast, the results from a case-control study including 74 infants with spina bifida and 87 non-malformed controls indicated that variants in *PAX3* were not strong risk factors for human spina bifida [20]. Therefore, exploring mechanisms other than coding sequence variants, such as methylation modification, in *PAX3* may provide novel insight into the etiology of human NTDs.

We hypothesized that aberrant DNA methylation of *Pax3* plays a role in the formation of NTDs. To test this hypothesis, we first compared the difference in methylation levels of CpG sites within *PAX3* using genomic methylation array data with DNA from neural tissues of NTD cases and non-malformed controls. The methylation status of the CpG sites that were found to differ was then validated in a larger NTD case-control population. We tested whether there was any correlation between CpG site methylation levels in fetal neural tissues and maternal serum concentrations of polycyclic aromatic hydrocarbons (PAHs), a class of ubiquitous environmental pollutants that have been shown to be associated with the risk for NTDs in epidemiological studies [21]. Finally, we assessed the methylation level and gene expression of *Pax3* in neural tissues from mouse embryos exposed in utero to benzo[a]pyrene (BaP), a widely studied PAH that induces NTDs [22]. In addition, we assayed markers of oxidative stress in fetal mice to further explore the possible mechanisms by which BaP might affect methylation regulation.

## Results

### Methylation of *PAX3* gene in genomic microarray

A detailed description on the genome-wide methylation results obtained from Infinium HumanMethylation450 BeadChip (HM450K), using DNA isolated from neural tissues from ten NTD cases and eight unrelated non-malformed controls can be found elsewhere [23]. In brief, out of 485,199 CpG sites across the entire genome, 23,294 (4.8%) were differentially methylated between cases and controls. Of the differentially methylated CpG sites, 12,383 (53.2%) were significantly hypermethylated and 10,911 (46.8%) were significantly hypomethylated in NTD cases when compared to controls. The characteristics of the NTD cases and controls in phase 1 are presented in Additional file 1: Table S1.

**Fig. 1:**
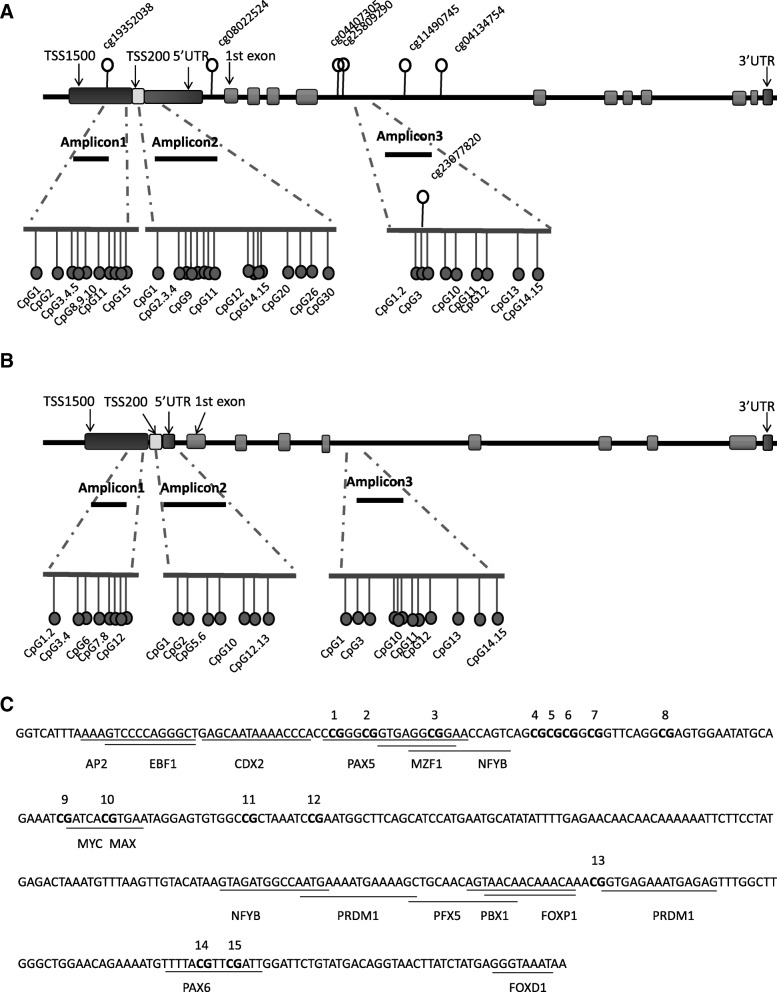
Schematic diagram showing the location of *PAX3* region analyzed. **a** Locations of the CpG sites detected in phase 1 and phase 2 of human methylation analysis. The differentially methylated CpG units identified by HM450K were indicated as white dots that above the black line (cg19352038, cg08022524, cg04407305, cg25809290, cg23077820, cg11490745, and cg04134754). In phase 2, three DNA amplicons were developed to validate the differentially methylated region detected in phase 1; amplicon 1 covered 10 analytical CpG sites located in the TSS1500 region of *PAX3*; amplicon 2 covered 16 analytical CpG sites located between TSS200 and 5′UTR region; amplicon 3 covered 9 analytical CpG sites located between the 4th and 5th exon. All the CpG sites detected in replication phase were indicated as black dots under the line. **b** Locations of the *Pax3* CpG sites detected in mouse methylation analysis. Three DNA amplicons were developed as in human study. Amplicon 1 covered 8 analytical CpG sites located in the TSS1500 region of *Pax3*; amplicon 2 covered 7 analytical CpG sites which located between TSS200 and 5′UTR region; amplicon 3 covered 12 analytical CpG sites located between the 4th and 5th exon. **c** Schematic diagram of interest indicating putative transcription factor-binding sties in amplicon 3 of human *PAX3* gene in intron 4. CpG sites predicted by JASPAR (predictive value > 8) to bind transcription factors are described under the sequence. CpG site 3 was the same as cg23077820 identified differentially by HM450K in phase 1. All the CpG sites except CpG site 12 were differentially methylated between NTDs and controls by Sequenom EpiTYPER in phase 2

In the *PAX3* gene, the focus of this study, a total of 54 CpG sites were extracted from the HM450K array data, of which 47 CpG sites (87.0%) were found to be hypermethylated. And 7 out of the 47 CpG sites exhibited statistically greater methylation in NTD fetuses than in controls (Additional file 2: Table S2). Analysis of the genomic location of the seven significantly hypermethylated CpG sites showed that one CpG was located at TSS1500, one in the 5′UTR, and the remaining five CpGs were within the body of the gene (Fig. 1a).

### Validation of *PAX3* methylation in an independent cohort of NTD cases and controls

**Fig. 2:**
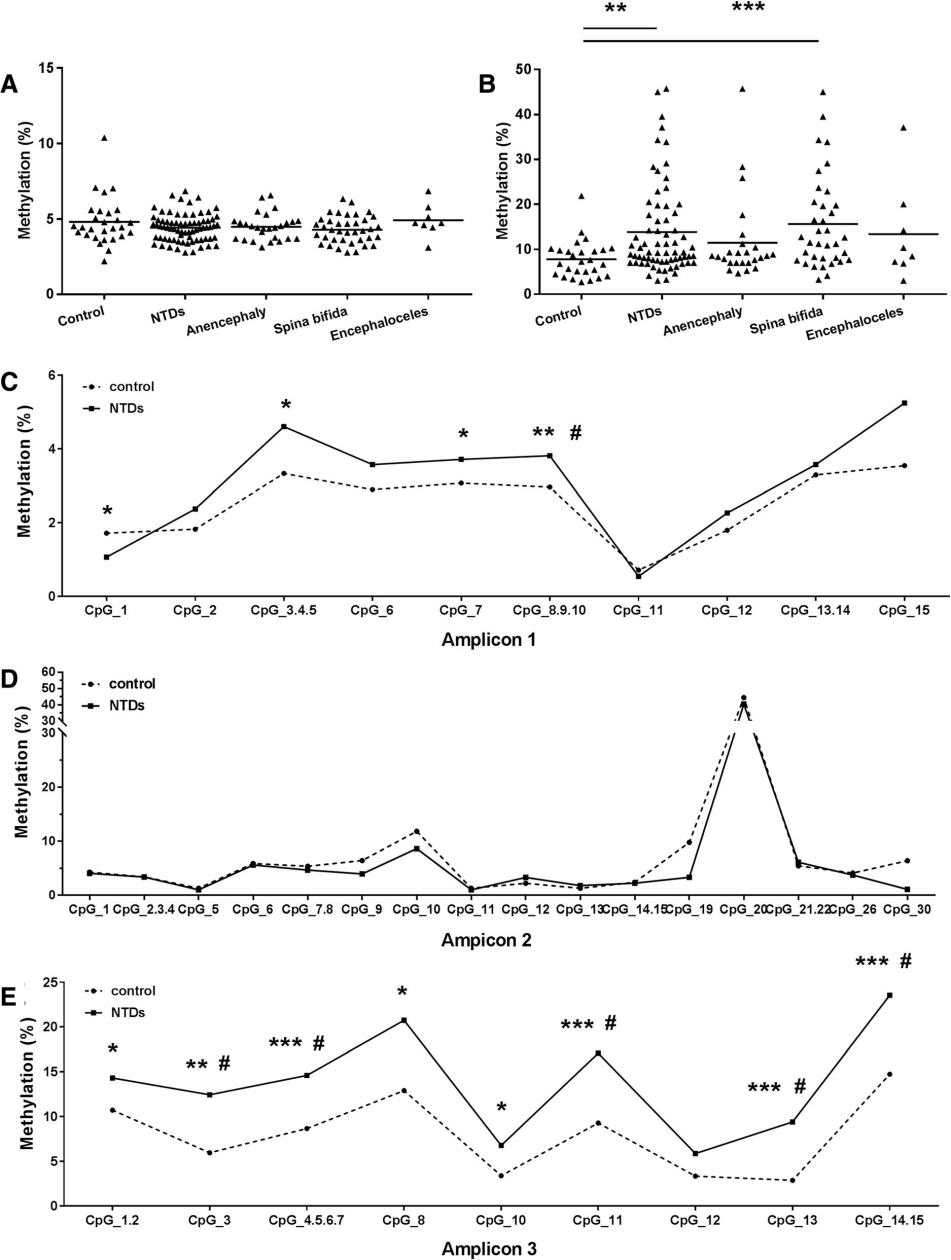
*PAX3* methylation pattern assayed by Sequenom EpiTYPER in NTD cases and controls. **a** Mean methylation level of *PAX3* promoter region (amplicon 1 and amplicon 2) between NTD cases and controls. **b** Mean methylation level of *PAX3* body region (amplicon 3) between NTD cases and controls. **c**–**e** Methylation levels for each CpG site between NTD cases and controls in promoter (**c**, **d**) and body region (**e**). TSS1500, TSS200, 5′URT, and the 1st exon were defined as the promoter region of *PAX3* gene in this study. The significance of differences was calculated using Independent t-test. *P <  0.05, **P <  0.01, ***P <  0.001, compared with control group. ^#^*P* <  0.05, compared with control group after FDR adjustment

In order to validate these findings, 73 NTD cases and 29 controls were used to examine the differentially methylated regions of *PAX3* identified in phase 1 using Sequenom EpiTYPER. The demographic characteristics of the subjects are described in Table 1. Three DNA amplicons were developed that cover 35 CpGs (Fig. 1a). In the present study, TSS1500, TSS200, 5′UTR, and the 1st exon were defined as the promoter region of *PAX3* gene. As shown in Fig. 2a, no significant difference in overall mean methylation levels in the promoter region of *PAX3* was observed between NTD cases and controls. However, NTD samples exhibited significantly higher DNA methylation levels (13.9 ± 10.1%) than control samples (7.8 ± 4.2%) in the gene body region (*P* = 0.003) (Fig. 2b). Furthermore, within the gene body, all CpG sites except CpG_12 showed significantly higher levels of methylation in the NTD samples compared to controls (Fig. 2e). After Benjanmini-Hochberg correction, methylation levels in five of the nine CpG sites were still significantly higher in NTD cases.

**Table 1:** Characteristics of NTD cases and controls in phase 2 for methylation assay

Characteristic	Control (*N* = 29)^a^	Case (*N* = 73)^a^	*P* value^b^
Maternal age (years)			0.703
< 25	14 (50.0)	30 (41.1)	
25–29	6 (21.4)	20 (27.4)	
≥ 30	8 (28.6)	23 (31.5)	
BMI (kg/m^2^)			0.595
< 18.5	2 (6.9)	6 (8.6)	
18.5–27.9	22 (75.9)	57 (81.4)	
≥ 28	5 (17.2)	7 (10.0)	
Maternal education			0.005
Primary or lower	4 (13.8)	9 (12.5)	
Junior high	12 (41.4)	52 (72.2)	
High school or above	13 (44.8)	11 (15.3)	
Occupation			< 0.001
Farmer	14 (48.3)	61 (87.1)	
Non-farmer	15 (51.7)	9 (12.9)	
Previous birth defects history			0.200
Yes	0	4 (5.6)	
No	28 (100)	67 (94.4)	
Gravidity			0.219
1	17 (58.6)	32 (45.1)	
≥ 2	12 (41.4)	39 (54.9)	
Parity			0.060
1	18 (72.0)	32 (50.0)	
≥ 2	7 (28.0)	32 (50.0)	
Periconceptional folic acid supplementation			< 0.001
Yes	4 (14.3)	42 (60.0)	
No	24 (85.7)	28 (40.0)	
Cold or fever			0.059
Yes	5 (17.9)	27 (37.5)	
No	23 (82.1)	45 (62.5)	
Active or passive smoking			0.210
Yes	15 (55.6)	29 (41.4)	
No	12 (44.4)	41 (58.6)	
Drinking			0.940
Yes	17 (65.4)	47 (66.2)	
No	9 (34.6)	24 (33.8)	
Gestational age (weeks)			0.023
< 28	14 (48.3)	52 (72.2)	
28–36	7 (24.1)	14 (19.4)	
> 36	8 (27.6)	6 (8.3)	
Fetal sex			0.129
Male	17 (58.6)	28 (41.8)	
Female	12 (41.4)	39 (58.2)	

^a^Data were presented in number (percentage). Total number may not be equal to the total of cases or controls due to missing or unknown data

^b^Cases and controls were compared by Pearson’s χ^2^ test, or Fisher’s exact test if any cell expectation was less than 5

The relationship between hypermethylation of *PAX3* and the risk of NTDs was examined. Methylation levels were categorized according to the median methylation level of the controls. As shown in Table 2, a higher level of methylation in the gene body region was associated with 6.24-fold increased risk for NTDs (95% CI 1.30–29.97).

**Table 2:** Risks of NTDs associated with methylation level of *PAX3* gene in fetal neural tissues

	All NTDs		Anencephaly		Spina bifida	
	OR^a^ (95% CI)	*P* value	OR^a^ (95% CI)	*P* value	OR^a^ (95% CI)	*P* value
Promoter region	0.69 (0.19, 2.45)	0.690	0.68 (0.15, 3.17)	0.683	0.57 (0.11, 2.98)	0.502
Amplicon_1_CpG_1	0.58 (0.17, 2.04)	0.398	0.44 (0.09, 2.18)	0.444	0.41 (0.07, 2.36)	0.414
Amplicon_1_CpG_3.4.5	*4*.*24* (*1*.*14*, *15*.*77*)	*0*.*031*	3.26 (0.56, 18.85)	0.187	4.22 (0.87, 20.56)	0.075
Amplicon_1_CpG_7	2.49 (0.73, 8.55)	0.146	1.28 (0.26, 6.36)	0.760	*5*.*86* (*1*.*18*, *29*.*10*)	*0*.*031*
Amplicon_1_CpG_8.9.10	3.11 (0.87, 11.09)	0.080	3.04 (0.48, 19.36)	0.239	*6*.*57* (*1*.*23*, *35*.*11*)	*0*.*028*
Body region	*6*.*24* (*1*.*30*, *29*.*97*)	*0*.*022*	4.30 (0.71, 26.25)	0.114	*10*.*76* (*1*.*06*, *109*.*45*)	*0*.*045*
Amplicon_1_CpG_1.2	2.87 (0.81, 10.17)	0.103	2.34 (0.52, 10.51)	0.266	*25*.*16* (*1*.*57*, *404*.*30*)	*0*.*023*
Amplicon_1_CpG_3	*4*.*34* (*1*.*12*, *16*.*78*)	*0*.*034*	4.12 (0.78, 21.89)	0.096	5.15 (0.81, 32.68)	0.082
Amplicon_1_CpG_4.5.6.7	1.42 (0.40, 5.12)	0.588	1.08 (0.25, 4.72)	0.917	3.35 (0.44, 25.28)	0.241
Amplicon_1_CpG_8	0.94 (0.16, 5.54)	0.945	0.40 (0.19, 20.06)	0.997	3.98 (0.19, 82.64)	0.372
Amplicon_1_CpG_10	4.83 (0.99, 23.55)	0.052	2.04 (0.33, 12.65)	0.443	*16*.*95* (*1*.*74*, *165*.*25*)	*0*.*015*
Amplicon_1_CpG_11	2.19 (0.61, 7.80)	0.227	0.72 (0.13, 3.90)	0.698	5.30 (0.65, 43.29)	0.120
Amplicon_1_CpG_13	3.05 (0.83, 11.15)	0.092	1.08 (0.21, 5.55)	0.930	*5*.*23* (*1*.*00*, *27*.*75*)	*0*.*050*
Amplicon_1_CpG_14.15	*7*.*52* (*1*.*36*, *41*.*58*)	*0*.*021*	6.23 (0.86, 45.03)	0.070	6.65 (0.68, 65.51)	0.104

^a^OR was adjusted by maternal education, occupation, periconceptional folic acid use and fetal sex, gestational age. ORs were in italic when *P* value < 0.05

### Correlation between PAH concentrations in maternal serum and *PAX3* methylation in fetal neural tissues

A previous study from our team found that higher PAH concentrations in maternal serum were associated with an increased risk for NTDs [24]. We therefore conducted a correlation analysis between PAH concentrations in maternal serum and *PAX3* methylation levels in fetal neural tissues (*N* = 51 mother-fetus pairs) to further our understanding of the potential relationship of PAH exposure, gene methylation, and NTDs risk. Notably, a significant positive correlation was found between mean methylation levels in the *PAX3* gene body and median concentrations of high-molecular-weight PAHs in maternal serum (*r* = 0.310. *P* = 0.027) (Additional file 3: Table S3).

### Disturbed methylation of *Pax3* in mouse embryos exposed to BaP

**Fig. 3:**
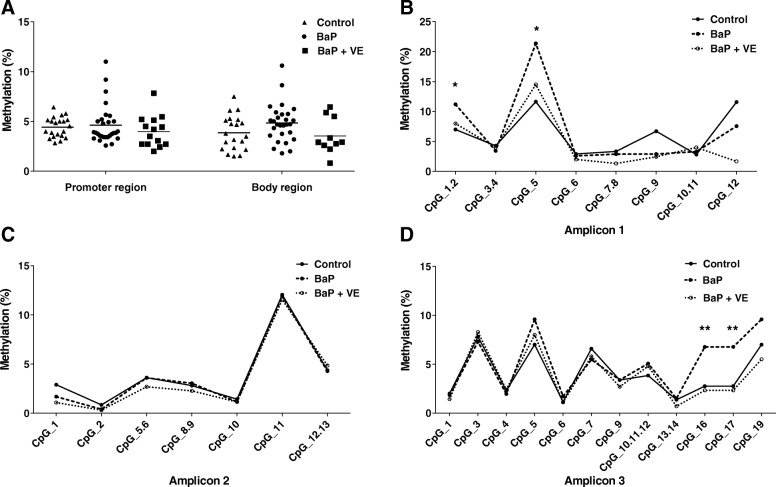
The effects of BaP and vitamin E on *Pax3* methylation in E10.5 mouse embryos. **a** Mean methylation level of promoter region (amplicon 1 and amplicon 2) and body region (amplicon 3) within *Pax3*. **b**–**d** Methylation level for specific CpG sites in promoter region (**b**, **c**) and body region (**d**) among control group, BaP-treated group and vitamin E co-supplementation group (*n* = 24–28). TSS1500, TSS200, 5′URT, and the 1st exon were defined as the promoter region of *Pax3* gene in this study. The significance of differences was calculated using ANOVA. *P < 0.05, **P < 0.01, ***P < 0.001, compared with control group. *VE* vitamin E

We further investigated the finding of a correlation between maternal serum PAH concentrations and *PAX3* hypermethylation in humans by utilizing a BaP-induced NTD mouse model. Three amplicons developed according to the mouse genome were used (Fig. 1b). The body region of *Pax3* showed a trend toward higher levels of methylation in the BaP-treated group compared with controls (Fig. 3a), but this did not reach statistical significance. Furthermore, in terms of specific CpG sites, two significantly hypermethylated CpG sites within the promoter and two within the body region of *Pax3* were respectively detected in the BaP-exposed mouse fetuses when compared to the non-exposed fetuses (Fig. 3b–d).

### The role of oxidative stress in BaP-related disturbed *Pax3* methylation and expression

**Fig. 4:**
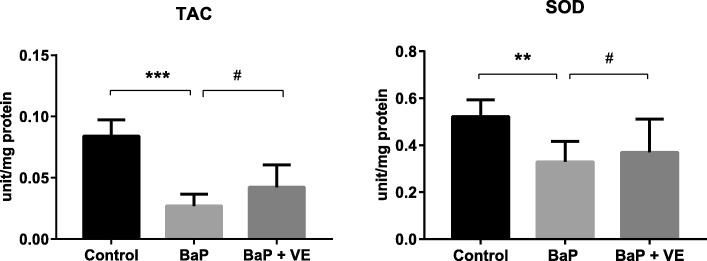
The effects of BaP and vitamin E on TAC and SOD activity in E10.5 mouse embryos. Data were expressed as mean ± SD (*n* = 8–10). *P < 0.05, **P < 0.01, ***P < 0.001, compared with control group; ^#^*P* < 0.05, ^##^*P* < 0.01, ^###^*P* < 0.001 compared with vitamin E supplemented group. *VE* vitamin E

**Fig. 5:**
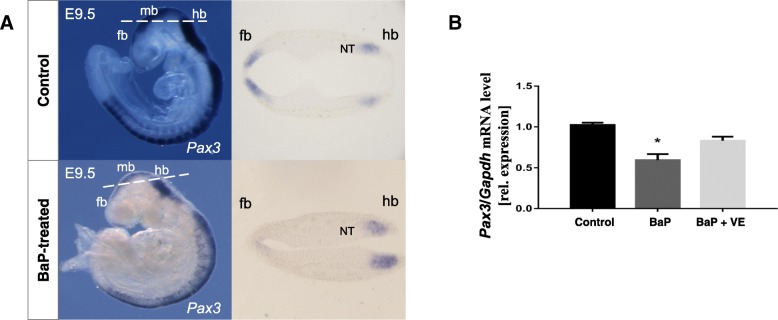
The Effects of BaP and vitamin E on *Pax3* gene expression in mouse embryos. **a** Whole mount in situ hybridization of *Pax3* in E9.5 embryos. In control embryos, *Pax3* mRNA was detected in the dorsal neural tube and somites as expected, while less gene expression was seen in the forebrain and midbrain of embryos exposed to BaP. The dashed lines on the whole mount panels indicate the orientation of respective sections. **b** Relative quantitative real-time PCR of *Pax3* mRNA from E10.5 embryos exposed to BaP and co-supplemented with vitamin E. Values were normalized against *Gapdh* and represented as mean ± SE (*n* = 5–8). *P < 0.05, compared with control group. *fb* forebrain, *mb* midbrain, *hb* hindbrain, *NT* neuroepithelium, *VE* vitamin E

Since BaP exposure was hypothesized to favor the generation of reactive oxygen species (ROS) in the embryos, we inferred that oxidative stress may get involved in the aberrant *Pax3* methylation induced by BaP. As shown in Fig. 4, compared to the control group, the levels of total antioxidant capacity (TAC) and the activity of superoxide dismutases (SODs) were decreased. Real-time PCR and whole mount in situ hybridization showed that BaP exposure significantly decreased the transcription of *Pax3* (*P* = 0.008) (Fig. 5). After co-supplemented with vitamin E, a commonly used antioxidant, the repressed levels of TAC and SODs were partly restored (Fig. 4). Coincidentally, the hypermethylation of *Pax3* in specific CpG sites induced by BaP treatment was recovered and *Pax3* expression was also normalized by vitamin E supplementation (Figs. 3 and 5), which suggested a causal effect of oxidative stress on both *Pax3* methylation and expression.

## Discussion

*Pax3* is a key gene, encoding a transcription factor required for neural tube closure [17]. In this study, we evaluated the methylation status of *PAX3* in a two-phase design study to examine whether there is a potential role of *PAX3* methylation in the development of human NTDs. Differentially hypermethylated CpG sites were found in the promoter and gene body region within *PAX3* in the neural tissues of NTD cases. Association analysis showed that a higher methylation level in the gene body region was associated with an elevated risk for NTDs. Moreover, PAH concentrations in maternal serum, which were known to be associated with increased risk of NTDs, were positively correlated to methylation levels at several CpG sites. In the BaP-induced NTD mouse model, hypermethylation of *Pax3* gene and suppressed gene expression were observed in embryos with BaP treatment, along with reduced TAC level. We previously showed that vitamin E supplementation could decrease the rate of NTDs and alleviate oxidative stress in BaP exposed embryos [22]. Here, we further demonstrated that vitamin E mitigated the shifts in *Pax3* methylation and gene expression.

Studies on the role of the *Pax3* in NTDs have mostly focused on gene depletion or loss of function mutants [17]. In mice, mutations in *Pax3* give rise to the *Splotch* (*Sp*) phenotype, which includes exencephaly, spina bifida, and neural crest abnormalities in homozygous mutant embryos [25, 26]. The human *PAX3* gene exhibits high homology with mouse *Pax3*. Mutations within the human *PAX3* gene have been found in Waardenburg syndromes, a condition which is occasionally associated with NTDs [18]. A previous study identified two spina bifida patients who had small interstitial chromosomal deletions involving *PAX3* [27]. Exon sequencing of *PAX3* in 114 cases with spina bifida also identified two common variants; however, without unaffected individuals, the influence of these variants on the risk of spina bifida could not be determined [28]. To the contrary, the screening results from 74 spina bifida cases and 87 control infants suggested that variant in *PAX3* was not a major contributor to the overall burden of NTDs at population level [20]. Recently, evidence from animal studies suggested that aberrant methylation of *Pax3* was involved in the development of NTDs induced by hyperglycemia [29, 30]. However, to date, no study investigating the role of *PAX3* methylation in the etiology of human NTDs has been reported. In our study, we analyzed the methylation status of *PAX3* gene in neural tissues from human fetuses, and our results showed that the methylation level in *PAX3* was significantly higher in NTD cases than in non-malformed controls.

Unexpectedly, in our study, compared with promoter region, the body region of *PAX3* was more consistently hypermethylated in NTD cases and the differentially methylated region was mainly located in intron 4. Our results imply that a methylation change in the body region of *PAX3* may be an epigenetic component of human NTDs, which is reminiscent with findings for the *HOXB7* gene in a myelomeningocele (spina bifida) case-control study [15]. In the latter study, a genome-wide methylation assay found three CpGs in *HOXB7* gene body to be hypomethylated in myelomeningocele patients when compared to controls, and these were further verified in a larger population, using the Sequenom EpiTYPER platform. Studies on *GRHL3* and *SOX18* also reported that altered methylation within the gene body was associated with the risk for NTDs [14, 31].

It is generally accepted that DNA methylation changes in the promoter and gene body regions may have differential effects on gene expression. DNA methylation in promoter regions is usually negatively associated with gene expression, whereas in the gene body, the impact is not consistent, with both positive and negative impacts having been reported [32, 33]. In the present study, a negative correlation between gene body DNA methylation and transcription level of *Pax3* was observed in mouse embryos, which was in line with the finding in hyperglycemia-induced NTDs [29]. The underlying mechanisms of gene body methylation in regulating gene expression have not been well understood. Recently, increasing evidence demonstrating the role of DNA methylation in alternative splicing regulation has been reported, which is essential for providing tissue-specific features for some genes [34]. DNA methylation is normally more abundant in exons compared to the flanking introns, which is a marker for distinguishing exons from introns. Previous studies proposed that the change of exon methylation levels would affect the recognition of exons while splicing [35]. It is thus reasonable to hypothesize that the methylation status of intron might also be crucial for alternative splicing. Further, *cis*-acting element has been recognized in intron, which could regulate gene expression [34]. Within intron 4 of *PAX3*, a number of transcription factors were predicted to bind at several CpG sites examined in the present study, with high predictive values (predictive value > 8, JASPAR, Fig. 1c), which might be responsible for the observed repressed gene expression. However, more research is necessary to examine this point in detail.

*PAX3* hypermethylation by itself is not likely to be a sole cause for NTDs but rather be part of a complex combination of environmental and epigenetic risk factors. Our previous epidemiological studies have suggested that maternal exposure to PAHs was associated with an elevated risk of NTDs in the offspring [21, 24]. Cohort studies reported that prenatal PAH exposure was associated with lower global methylation and hypermethylation of *interferon* γ in umbilical cord white blood cells [36, 37]. Several toxicological studies have also suggested that BaP exposure could disrupt DNA methylation status [38, 39]. Importantly, in the present study, we found that differentially methylated CpG sites in *PAX3* gene in fetal neural tissues were positively correlated with PAH concentrations in maternal serum. In line with these findings in human subjects, our mouse experiment showed that the methylation level of *Pax3* was elevated after BaP treatment, indicating that the presence of BaP impacts *Pax3* methylation regulation. These findings support the hypothesis that hypermethylation of *Pax3* gene is involved in abnormal closure of neural tube secondary to PAH exposure.

In considering how BaP exposure could affect methylation, accumulating evidence has demonstrated that oxidative stress in response to various environmental insults or maternal dietary factors is responsible for aberrant DNA methylation [40–43]. In our study, hypermethylation of *Pax3* and repressed gene expression, along with reduced TAC level, were observed in BaP-treated mouse embryos. Co-administration with vitamin E could rescue NTDs induced by BaP, partly normalized the TAC level, and attenuate the hypermethylation of *Pax3* and the repressed gene expression. All these findings support the postulation that the oxidative stress may be causally involved in aberrant DNA methylation in the BaP-treated group, which might be the underlying mechanism for the development of NTDs caused by BaP. Consistent with this idea, a previous investigation revealed that epigallocatechin gallate, which is the major polyphenol in green tea and is believed to act as an antioxidant, could block hypermethylation of several neural tube closure essential genes induced by maternal diabetes, including *Pax3* [30]. Although it is not precisely known, previous investigations proposed that oxidative stress could influence the synthesis of S-adenosylmethionine [44], the expression of DNA methyltransferase [45], and the activity of the ten eleven translocation enzymes [46], which are all critical for DNA methylation. On the other hand, epigenetic regulation may also contribute to impairment of antioxidant gene expression [47]. In this regard, the ROS production and epigenetic regulation establish an interconnected cycle, which would amplify external factors toward the progression of pathological disorders.

DNA methylation is considered tissue-specific and therefore appropriate biological samples for methylation study are crucial. One advantage of our study is that neural tissues from fetuses were used for assessment of methylation, which makes our methylation study more relevant to the outcome, as compared to those that used blood DNA as the surrogate [15]. The limitation is that we did not investigate the expression of *PAX3* gene in our human subjects, as fresh neural tissue samples of terminated NTD cases for RNA assay are extremely difficult to collect. Although our mouse experiment may provide a clue, additional studies are needed to correlate the methylation levels of the *PAX3* gene body with *PAX3* gene or protein expression values. Another limitation is that more case mothers reported folic acid supplementation than control mothers, which might cause the differences in methylation levels between the two groups. However, when analyses were performed by folic acid supplementation status in the case group, no differences in the methylation level of *PAX3* were found between those with or those without folic acid use (Additional file 4: Figure S1), suggesting that folic acid has no impact on methylation level in the present study.

## Conclusions

In conclusion, we found that hypermethylation of *Pax3* and the downregulation of the gene may be important events in the development of NTDs during embryogenesis. A relationship exists between higher maternal serum PAH concentrations and *PAX3* hypermethylation in fetal neural tissues, which is further supported by our mouse experiments. In addition, oxidative stress may be involved in the process of environmental exposure and methylation modification. Our study provides novel evidence on the interaction between genetic and environmental factors in the etiology of NTDs.

## Methods

### Study subjects

The human subjects were recruited from rural counties in Shanxi province of northern China (Pinding, Zezhou, Xiyang, Shouyang, and Taigu), where NTD prevalence is among the highest in the world [48]. As described in more detail in our previous report [48], NTD cases were terminated fetuses affected by an NTD; controls were terminated non-malformed fetuses. Information on maternal sociodemographic characteristics, lifestyle, and folic acid supplementation was collected through in-person interviews and by viewing medical records. Maternal venous blood samples were collected at pregnancy termination. Fetal spinal cord and brain tissues were collected by autopsy performed by experienced pathologists. All samples were stored at − 80 °C until assay. The study protocol was approved by the Institutional Review Board of Peking University, and written informed consent was obtained from all participating women.

### Experimental animals

ICR mice of 8–9 weeks old were used, as described previously [22]. Briefly, BaP (Sigma, USA) dissolved in corn oil was intraperitoneally administered into pregnant mice from E7 for four consecutive days (250–350 mg/kg). Mice in the vitamin E co-exposure group were fed with chow supplemented with the water-soluble (±)-α-tocopherol succinate form of vitamin E (Sigma, USA) beginning from E0.5 (0.125%, *w*/*w*) and treated with BaP from E7 (250 mg/kg). On E10.5, embryos were collected and carefully inspected for NTDs. All experimental procedures were approved by the Institutional Animal Care and Use Committee of Peking University (certificate no. LA2013-36).

### DNA methylation level analysis

DNA from neural tissues of human subjects was extracted with QIAamp DNA Mini Kit (QIAGEN, Hilden, Germany) and E10.5 mouse DNA was extracted from frozen neural tissues using TIANamp Genomic DNA Kit (TIANGEN Biotech, Beijing, China). Then, 500 ng of genomic DNA from each sample was bisulfite-treated with EZ DNA methylation kit (Zymo Research, CA, USA). The bisulfite conversion reaction was performed in duplicate for each sample to minimize potential bias caused by variable conversion efficiency, and pooled bisulfite-treated DNA was used for subsequent analysis. Human methylation study was performed in two phases. In phase 1, Infinium HumanMethylation450 BeadChip assay (450K, Illumina, San Diego, CA, USA) was used for genomic methylation assay of bisulfite-treated DNA from ten NTD cases and eight non-malformed controls, which has been described in detail in our previous study [23]. Methylation data of *PAX3* gene was extracted from the array data and used for validation in the next phase.

In phase 2, 73 NTD cases and 29 non-malformed control fetuses were included. The locations of the amplicons to target the aberrant CpG regions are shown in Fig. 1a. Bisulphite DNA was amplified by PCR and primers for the *PAX3* gene were designed using the online tool Epidesigner (www.epidesigner.com). Primer sequences are listed in Additional file 5: Table S4. After reverse transcription, fragmentation, and analysis on a mass spectrometer (Sequenom, Inc., San Diego, USA), EpiTYPER Analyzer software was used for translating mass signal patterns into quantitative DNA methylation levels of different CpG sites.

Methylation analysis of mouse neural tissues was assayed with the same methods performed in phase 2 of the human methylation study. The regions of gene sequence analyzed were the same as those in human *PAX3* gene and three amplicons were designed, with two in the promoter region and one in the body region, as shown in Fig. 1b. See Additional file 6: Table S5 for PCR primer sequences.

### PAHs analysis

PAH concentrations in maternal blood were determined with an Agilent 7890A-5975C gas chromatograph and mass spectrometer equipped with a HP-5MS capillary column (30 m × 0.25 mm × 0.25 μm), as described previously [24]. In the present study, 51 mother-fetus pairs were available for PAH-methylation correlation analysis.

### Oxidative stress assessment

The antioxidant capacity was determined by the reduced ferric reducing antioxidant power (FRAP) assay according to the manufacturer’s instructions (Beyotime Institute of Biotechnology, China) for determining the level of TAC of mouse embryos. The TAC aims to measure both small molecule and protein antioxidants, including polyphenols, flavonoids, vitamins, and enzymes like glutathione peroxidase and superoxide dismutase. Briefly, neural tissues from E10.5 embryo samples were homogenized in PBS. Freshly prepared FRAP reagent was warmed to 37 °C before use. Then, 5 μl of the diluted sample was added to 180 μl of the FRAP reagent. The absorbance of the mixture was measured at 593 nm using a Synergy 2 Multi-Mode Microplate Reader (BioTek, USA) after incubation for 4 min. The TAC of each sample was calculated from the standard curve constructed using FeSO_4_ solution, and the results were expressed as mmol/g protein, adjusted according to the protein concentration of the samples. All samples were loaded in duplicate. Protein concentration was determined using the BCA protein assay kit (Beyotime Institute of Biotechnology, China).

SOD activity was determined using the total superoxide dismutase assay kit with WST-8 (Beyotime Institute of Biotechnology, China). Briefly, 20 μl of sample (E10.5 embryo samples were homogenized in lysis buffer) was mixed with 160 μl of WST-8/enzyme working solution. Then, 20 μl of reaction triggering working solution was added. After incubation at 37 °C for 30 min, the absorbance was determined at 450 nm using a Synergy 2 Multi-Mode Microplate Reader (BioTek, USA). The SOD activity was expressed as U/mg protein.

### RNA isolation and real-time PCR

RNA was isolated from E10.5 embryos neural tissues using Trizol (Invitrogen); genomic DNA was removed by DNase I digestion (DNA-free, Ambion) and then reverse-transcribed using random hexamers (Superscript VILO cDNA synthesis kit). The abundance of mRNA of *Pax3* were analyzed using real-time PCR (iTaq™ Universal SYBR Green Supermix, BioRad) on a 7500 Fast Real Time PCR system (Applied Biosystems), with each sample analyzed in triplicate. Primers are listed in Additional file 7: Table S6. Relative quantification of gene expression level was normalized according to the *Gapdh* gene expression.

### Whole mount in situ hybridization

Whole mount in situ hybridization on E9.5 control embryos and embryos treated with BaP was performed according to the procedures described by Yun et al. [49]. *Pax3* probe was cloned by real-time PCR into pGEM-T (Promega) and used to generate digoxygenin-labeled cRNA probes by reverse transcription using T7 RNA polymerase (Roche). For detection, anti-digoxigenin-AP antibody (1:2000, Roche) in 1% sheep serum was used and incubated overnight. Color detection was carried out using NBT/BCIP developing solution (Roche) in NTMT. After color development, embryos were imaged with a DFC490 camera (Leica), and then embedded. Sections of 40 μm thickness were obtained using a vibratome.

### Statistical analyses

In the human subject study, differences in proportions of population characteristics between NTD cases and controls were examined with Pearson’s χ^2^ test. Independent *t* test was performed to evaluate the difference in methylation of CpGs between NTDs and controls in phase 1, and adjusted for multiple testing with the Benjamini-Hochberg false discovery rate (FDR) methods. In phase 2, Shapiro-Wilk test was used to examine the distribution of methylation values of NTD cases and controls, and independent samples *t* test was used to identify CpG sites that were differentially methylated between cases and controls. Odds ratio (OR) was calculated by logistic regression to evaluate the association between higher methylation levels of *PAX3* with the risk of NTDs, adjusting for maternal education, occupation, periconceptional folic acid use, fetal sex, and gestational age. Correlation between differentially methylated CpG sites in neural tissues and PAH concentrations in maternal serum was analyzed with Spearman’s correlation analysis. In the mouse study, data on methylation level of *Pax3* gene, oxidative stress markers, and the abundance of mRNA were presented as mean ± SE (SD). A one-way analysis of variance (ANOVA) followed by LSD (equal variances assumed) or Dunnett’s T3 (equal variances not assumed) was used for testing the differences between groups. A two-tail *P* value of < 0.05 was considered statistically significant. Statistical analyses were conducted using SPSS 18.0.

## Additional files

Additional file 1: **Table S1**. Characteristics of NTD cases and controls in phase 1 for methylation assay. (DOCX 18 kb)

Additional file 2: **Table S2**. Methylation of *PAX3* gene in phase 1 using the HumanMethylation450 BeadChip assay. (DOCX 24 kb)

Additional file 3: **Table S3**. Correlation analysis of DNA methylation of *PAX3* in fetal neural tissues and PAH concentrations in maternal serum (*N* = 51 mother-fetus pairs). (DOCX 22 kb)

Additional file 4: **Figure S1**. *PAX3* methylation pattern assayed by Sequenom EpiTYPER in NTD cases with and without folic acid supplementation. (DOCX 148 kb)

Additional file 5: **Table S4**. The PCR primer sequences in Sequenom EpiTYPER sequencing. (DOCX 15 kb)

Additional file 6: **Table S5**. The PCR primer sequences for mouse methylation analysis. (DOCX 15 kb)

Additional file 7: **Table S6**. Sequences of primers for real-time PCR in mouse study. (DOCX 15 kb)

## Abbreviations

BaP: Benzo[a]pyrene
FDR: False discovery rate
FRAP: Ferric reducing antioxidant power
HM450K: HumanMethylation450 BeadChip
NTDs: Neural tube defects
PAH: Polycyclic aromatic hydrocarbon
ROS: Reactive oxygen species
SOD: Superoxide dismutase
TAC: Total antioxidant capacity
