# Supplementary material for: Aberrant methylation of Pax3 gene and neural tube defects in association with exposure to polycyclic aromatic hydrocarbons
Source: Clin Epigenetics. 2019 Jan 21;11:13. doi: 10.1186/s13148-019-0611-7 (PMC6341549; doi:10.1186/s13148-019-0611-7)
Supplement: Supplementary file 1 — Table S1. Characteristics of NTD cases and controls in phase 1 for methylation assay. (DOCX 18 kb) [file 13148_2019_611_MOESM1_ESM.docx]

**Table S1.** Characteristics of NTD cases and controls in phase 1 for methylation assay

| Characteristic | Control (N = 8) ^a^ | Case (N = 10) ^a^ | *P* value ^b^ |
| --- | --- | --- | --- |
| Maternal age (y) |  |  | 0.596 |
| < 25 | 3 (42.9) | 6 (60.0) |  |
| 25 - 29 | 1 (14.3) | 2 (20.0) |  |
| ≥ 30 | 3 (42.9) | 2 (20.0) |  |
| BMI (kg/m^2^) |  |  | 0.800 |
| < 18.5 | 0 | 0 |  |
| 18.5 - 27.9 | 6 (75.0) | 8 (80.0) |  |
| ≥ 28 | 2 (25.0) | 2 (20.0) |  |
| Maternal education |  |  | 0.024 |
| Primary or lower | 2 (25.0) | 2 (20.0) |  |
| Junior high | 2 (25.0) | 8 (80.0) |  |
| High school or above | 4 (50.0) | 0 |  |
| Occupation |  |  | 0.003 |
| Farmer | 3 (37.5) | 10 (100) |  |
| Non-farmer | 5 (62.5) | 0 |  |
| Previous birth defects history |  |  | 0.090 |
| Yes | 0 | 3 (30.0) |  |
| No | 8 (100) | 7 (70.0) |  |
| Gravidity |  |  | 0.596 |
| 1 | 5 (62.5) | 5 (50.0) |  |
| ≥ 2 | 3 (37.5) | 5 (50.0) |  |
| Parity |  |  | 0.949 |
| 1 | 4 (57.1) | 5 (55.6) |  |
| ≥ 2 | 3 (42.9) | 4 (44.4) |  |
| Periconceptional  folic acid supplementation |  |  | 0.005 |
| Yes | 1 (12.5) | 8 (80.0) |  |
| No | 7 (87.5) | 2 (20.0) |  |
| Cold or fever |  |  | 0.196 |
| Yes | 1 (12.5) | 4 (40.0) |  |
| No | 7 (87.5) | 6 (60.0) |  |
| Active or passive smoking |  |  | 0.387 |
| Yes | 4 (50.0) | 3 (30.0) |  |
| No | 4 (50.0) | 7 (70.0) |  |
| Drinking |  |  | 0.250 |
| Yes | 7 (87.5) | 10 (100) |  |
| No | 1 (12.5) | 0 |  |
| Gestational age (weeks) |  |  | 0.668 |
| < 28 | 4 (50.0) | 7 (70.0) |  |
| 28 - 36 | 1 (12.5) | 1 (10.0) |  |
| > 36 | 3 (37.5) | 2 (20.0) |  |
| Foetal sex |  |  | 0.772 |
| Male | 3 (37.5) | 4 (44.4) |  |
| Female | 5 (62.5) | 5 (55.6) |  |

^a^ Data were presented in number (percentage). Total number may not be equal to the total of cases or controls due to missing or unknown data. ^b^ Cases and controls were compared by Pearson's χ^2^ test, or Fisher's exact test if any cell expectation was less than 5.
