# Supplementary material for: Aberrant methylation of Pax3 gene and neural tube defects in association with exposure to polycyclic aromatic hydrocarbons
Source: Clin Epigenetics. 2019 Jan 21;11:13. doi: 10.1186/s13148-019-0611-7 (PMC6341549; doi:10.1186/s13148-019-0611-7)
Supplement: Supplementary file 2 — Table S2. Methylation of PAX3 gene in phase 1 using the HumanMethylation450 BeadChip assay. (DOCX 24 kb) [file 13148_2019_611_MOESM2_ESM.docx]

**Table S2.** Methylation of *PAX3* gene in phase 1 using the HumanMethylation450 BeadChip assay

| Probe ID ^a^ | Chr | Mapinfo | Case | Control | Diff | *P* value ^b^ | Region ^c^ |
| --- | --- | --- | --- | --- | --- | --- | --- |
| cg01954737 | 2 | 223164459 | 0.162 | 0.106 | 0.056 | 0.266 | TSS1500 |
| cg10035294 | 2 | 223164925 | 0.100 | 0.046 | 0.054 | 0.100 | TSS1500 |
| cg10544031 | 2 | 223164635 | 0.144 | 0.105 | 0.039 | 0.379 | TSS1500 |
| cg13496838 | 2 | 223164747 | 0.181 | 0.073 | 0.108 | 0.052 | TSS1500 |
| cg16529477 | 2 | 223164854 | 0.201 | 0.095 | 0.106 | 0.116 | TSS1500 |
| cg18077971 | 2 | 223164867 | 0.161 | 0.065 | 0.096 | 0.083 | TSS1500 |
| cg19352038 | 2 | 223164869 | 0.270 | 0.083 | 0.186 | **0.042** | TSS1500 |
| cg27480727 | 2 | 223164831 | 0.257 | 0.114 | 0.143 | 0.068 | TSS1500 |
| cg09424526 | 2 | 223163809 | 0.032 | 0.030 | 0.002 | 0.789 | TSS200 |
| cg15133719 | 2 | 223163804 | 0.044 | 0.036 | 0.008 | 0.367 | TSS200 |
| cg15703632 | 2 | 223163725 | 0.068 | 0.069 | -0.001 | 0.914 | TSS200 |
| cg20584905 | 2 | 223163784 | 0.042 | 0.036 | 0.005 | 0.190 | TSS200 |
| cg08022524 | 2 | 223162128 | 0.352 | 0.228 | 0.124 | **0.049** | 5’UTR |
| cg14265823 | 2 | 223163326 | 0.067 | 0.062 | 0.005 | 0.361 | 1st exon |
| cg15608397 | 2 | 223163573 | 0.087 | 0.069 | 0.018 | 0.133 | 1st exon |
| cg02245378 | 2 | 223161771 | 0.186 | 0.078 | 0.108 | 0.064 | Body |
| cg08431536 | 2 | 223161894 | 0.151 | 0.041 | 0.110 | 0.075 | Body |
| cg00926215 | 2 | 223162049 | 0.135 | 0.049 | 0.086 | 0.062 | Body |
| cg01790894 | 2 | 223162692 | 0.511 | 0.403 | 0.109 | 0.163 | Body |
| cg10416206 | 2 | 223162797 | 0.207 | 0.166 | 0.041 | 0.302 | Body |
| cg13767755 | 2 | 223162800 | 0.228 | 0.221 | 0.007 | 0.933 | Body |
| cg14093610 | 2 | 223162666 | 0.201 | 0.147 | 0.054 | 0.277 | Body |
| cg06916239 | 2 | 223162881 | 0.136 | 0.128 | 0.009 | 0.581 | Body |
| cg23546474 | 2 | 223163033 | 0.089 | 0.083 | 0.006 | 0.831 | Body |
| cg25596297 | 2 | 223162875 | 0.095 | 0.083 | 0.012 | 0.563 | Body |
| cg20969194 | 2 | 223163175 | 0.087 | 0.079 | 0.008 | 0.416 | Body |
| cg00461612 | 2 | 223158076 | 0.420 | 0.482 | -0.061 | 0.441 | Body |
| cg02347074 | 2 | 223155925 | 0.126 | 0.114 | 0.012 | 0.112 | Body |
| cg04134754 | 2 | 223134024 | 0.700 | 0.318 | 0.382 | **0.003** | Body |
| cg04407305 | 2 | 223157265 | 0.544 | 0.377 | 0.167 | **0.017** | Body |
| cg04688351 | 2 | 223154140 | 0.201 | 0.075 | 0.126 | 0.096 | Body |
| cg09329930 | 2 | 223135786 | 0.936 | 0.917 | 0.019 | 0.470 | Body |
| cg11454719 | 2 | 223155841 | 0.076 | 0.065 | 0.011 | 0.214 | Body |
| cg11490745 | 2 | 223151884 | 0.720 | 0.515 | 0.205 | **0.011** | Body |
| cg15775921 | 2 | 223156136 | 0.092 | 0.073 | 0.019 | 0.455 | Body |
| cg15889391 | 2 | 223155306 | 0.122 | 0.080 | 0.043 | 0.403 | Body |
| cg16563171 | 2 | 223156106 | 0.067 | 0.062 | 0.005 | 0.787 | Body |
| cg16661269 | 2 | 223103743 | 0.439 | 0.465 | -0.026 | 0.719 | Body |
| cg19307391 | 2 | 223121905 | 0.898 | 0.907 | -0.009 | 0.810 | Body |
| cg22989843 | 2 | 223154201 | 0.207 | 0.118 | 0.088 | 0.173 | Body |
| cg23077820 | 2 | 223154176 | 0.432 | 0.189 | 0.243 | **0.012** | Body |
| cg25809290 | 2 | 223156901 | 0.529 | 0.282 | 0.248 | **0.008** | Body |
| cg01879473 | 2 | 223160102 | 0.112 | 0.046 | 0.067 | 0.144 | Body |
| cg02120658 | 2 | 223160297 | 0.223 | 0.104 | 0.118 | 0.125 | Body |
| cg09678971 | 2 | 223160486 | 0.182 | 0.066 | 0.115 | 0.242 | Body |
| cg10601940 | 2 | 223159305 | 0.346 | 0.249 | 0.098 | 0.121 | Body |
| cg11912765 | 2 | 223159838 | 0.126 | 0.042 | 0.084 | 0.135 | Body |
| cg13495369 | 2 | 223160569 | 0.124 | 0.070 | 0.054 | 0.289 | Body |
| cg18406033 | 2 | 223159869 | 0.206 | 0.052 | 0.154 | 0.098 | Body |
| cg19987665 | 2 | 223159250 | 0.172 | 0.087 | 0.085 | 0.177 | Body |
| cg24621972 | 2 | 223160175 | 0.238 | 0.070 | 0.167 | 0.065 | Body |
| cg16978914 | 2 | 223158408 | 0.288 | 0.301 | -0.013 | 0.888 | Body |
| cg03293308 | 2 | 223065390 | 0.590 | 0.843 | -0.253 | 0.000 | 3'UTR |
| cg24829778 | 2 | 223066461 | 0.833 | 0.966 | -0.133 | 0.017 | 3'UTR |

^a^ Probe ID is identified according to HM450K. The nucleotide position is based on NCBI build 37/hg19. ^b^ *P* value is adjusted for multiple testing using the Benjamini-Hochberg FDR methods to control for the false discovery rate. ^c^ Region is defined relative to the nearest open reading frame: within 1500 (TSS1500) or 200 bp (TSS200) of a transcription start site, in the 5’ untranslated region (5’UTR), the first exon of a transcript (1st exon), and in the body of gene (Body) and the 3’ untranslated region (3’UTR). Chr: chromosome; Mapinfo: nucleotide position; diff: difference.
