# Supplementary material for: Aberrant methylation of Pax3 gene and neural tube defects in association with exposure to polycyclic aromatic hydrocarbons
Source: Clin Epigenetics. 2019 Jan 21;11:13. doi: 10.1186/s13148-019-0611-7 (PMC6341549; doi:10.1186/s13148-019-0611-7)
Supplement: Supplementary file 3 — Table S3. Correlation analysis of DNA methylation of PAX3 in fetal neural tissues and PAH concentrations in maternal serum (N = 51 mother-fetus pairs). (DOCX 22 kb) [file 13148_2019_611_MOESM3_ESM.docx]

**Table S3.** Correlation analysis of DNA methylation of *PAX3* in fetal neural tissues and PAH concentrations in maternal serum (N = 51 mother-fetus pairs)

|  | ΣL_PAHs ^a^ | | ΣH_PAHs ^a^ | | ΣT_PAHs ^a^ | |
| --- | --- | --- | --- | --- | --- | --- |
|  | *r* ^b^ | *P* value | *r* ^b^ | *P* value | *r* ^b^ | *P* value |
| Promoter region | 0.085 | 0.543 | 0.082 | 0.554 | 0.080 | 0.567 |
| Amplicon_1_CpG_1 | 0.336 | **0.016** | 0.256 | 0.070 | 0.320 | **0.022** |
| Amplicon_1_CpG_2 | 0.034 | 0.814 | -0.042 | 0.772 | 0.022 | 0.878 |
| Amplicon_1_CpG_3.4.5 ^c^ | -0.107 | 0.480 | -0.093 | 0.539 | -0.087 | 0.567 |
| Amplicon_1_CpG_6 | -0.143 | 0.317 | -0.144 | 0.314 | -0.148 | 0.299 |
| Amplicon_1_CpG_7 ^c^ | 0.217 | 0.126 | 0.239 | 0.091 | 0.253 | 0.073 |
| Amplicon_1_CpG_8.9.10 ^c^ | 0.048 | 0.742 | -0.003 | 0.986 | 0.022 | 0.880 |
| Amplicon_1_CpG_11 | -0.076 | 0.598 | -0.084 | 0.560 | -0.090 | 0.529 |
| Amplicon_1_CpG_12 | -0.220 | 0.122 | -0.191 | 0.179 | -0.199 | 0.162 |
| Amplicon_1_CpG_13.14 | 0.335 | **0.016** | 0.276 | **0.050** | 0.314 | **0.025** |
| Amplicon_1_CpG_15 | 0.095 | 0.544 | 0.021 | 0.892 | 0.071 | 0.650 |
| Amplicon_2_CpG_1 | -0.142 | 0.310 | -0.185 | 0.185 | -0.169 | 0.226 |
| Amplicon_2_CpG_2.3.4 | -0.006 | 0.969 | 0.009 | 0.947 | 0.001 | 0.994 |
| Amplicon_2_CpG_5 | -0.071 | 0.612 | 0.011 | 0.936 | -0.051 | 0.716 |
| Amplicon_2_CpG_6 | 0.119 | 0.396 | 0.068 | 0.629 | 0.099 | 0.482 |
| Amplicon_2_CpG_7.8 | -0.047 | 0.738 | 0.055 | 0.695 | -0.012 | 0.931 |
| Amplicon_2_CpG_9 | 0.070 | 0.660 | 0.076 | 0.634 | 0.071 | 0.653 |
| Amplicon_2_CpG_10 | -0.106 | 0.449 | -0.152 | 0.278 | -0.122 | 0.384 |
| Amplicon_2_CpG_11 | -0.071 | 0.612 | 0.011 | 0.936 | -0.051 | 0.716 |
| Amplicon_2_CpG_12 | -0.159 | 0.254 | -0.122 | 0.385 | -0.156 | 0.264 |
| Amplicon_2_CpG_13 | -0.113 | 0.422 | -0.110 | 0.431 | -0.101 | 0.470 |
| Amplicon_2_CpG_14.15 | 0.051 | 0.719 | 0.095 | 0.499 | 0.062 | 0.660 |
| Amplicon_2_CpG_19 | 0.018 | 0.901 | 0.063 | 0.653 | 0.027 | 0.848 |
| Amplicon_2_CpG_20 | 0.104 | 0.460 | 0.109 | 0.439 | 0.094 | 0.503 |
| Amplicon_2_CpG_21.22 | -0.077 | 0.604 | -0.165 | 0.262 | -0.104 | 0.483 |
| Amplicon_2_CpG_26 | -0.048 | 0.732 | -0.010 | 0.941 | -0.043 | 0.758 |
| Amplicon_2_CpG_30 | 0.025 | 0.859 | 0.089 | 0.526 | 0.045 | 0.748 |
| **Body region** | 0.216 | 0.128 | 0.310 | **0.027** | 0.242 | 0.088 |
| Amplicon_1_CpG_1.2 ^c^ | 0.117 | 0.430 | 0.180 | 0.220 | 0.123 | 0.404 |
| Amplicon_1_CpG_3 ^c^ | 0.267 | 0.064 | 0.316 | **0.027** | 0.275 | 0.056 |
| Amplicon_1_CpG_4.5.6.7 ^c^ | 0.162 | 0.273 | 0.226 | 0.123 | 0.162 | 0.271 |
| Amplicon_1_CpG_8 ^c^ | 0.252 | 0.122 | 0.331 | **0.039** | 0.265 | 0.103 |
| Amplicon_1_CpG_10 ^c^ | -0.015 | 0.921 | 0.180 | 0.231 | 0.043 | 0.775 |
| Amplicon_1_CpG_11 ^c^ | 0.139 | 0.331 | 0.220 | 0.120 | 0.164 | 0.249 |
| Amplicon_1_CpG_12 | -0.040 | 0.797 | 0.105 | 0.499 | 0.021 | 0.895 |
| Amplicon_1_CpG_13 ^c^ | 0.168 | 0.244 | 0.306 | **0.031** | 0.222 | 0.122 |
| Amplicon_1_CpG_14.15 ^c^ | 0.043 | 0.774 | 0.135 | 0.364 | 0.054 | 0.717 |

^a^ ΣL_PAHs is the sum of low-molecular-weight PAHs including acenaphthylene, acenaphthene, fluorene, phenanthrene, anthracene, fluoranthene and retene; ΣH_PAHs is the sum of high-molecular weight-PAHs including pyrene, benz[a]anthracene, chrysene, benzo[b]fluoranthene, benzo[k]fluoranthene and benzo[a]pyrene; ΣT_PAHs is the sum of all PAHs. ^b^ *r*, Spearman's correlation coefficient. ^c^ CpG sites were found to be hypermethylated in NTDs when compared to controls.
