# Supplementary material for: Aberrant methylation of Pax3 gene and neural tube defects in association with exposure to polycyclic aromatic hydrocarbons
Source: Clin Epigenetics. 2019 Jan 21;11:13. doi: 10.1186/s13148-019-0611-7 (PMC6341549; doi:10.1186/s13148-019-0611-7)
Supplement: Supplementary file 4 — Figure S1. PAX3 methylation pattern assayed by Sequenom EpiTYPER in NTD cases with and without folic acid supplementation. (DOCX 148 kb) [file 13148_2019_611_MOESM4_ESM.docx]

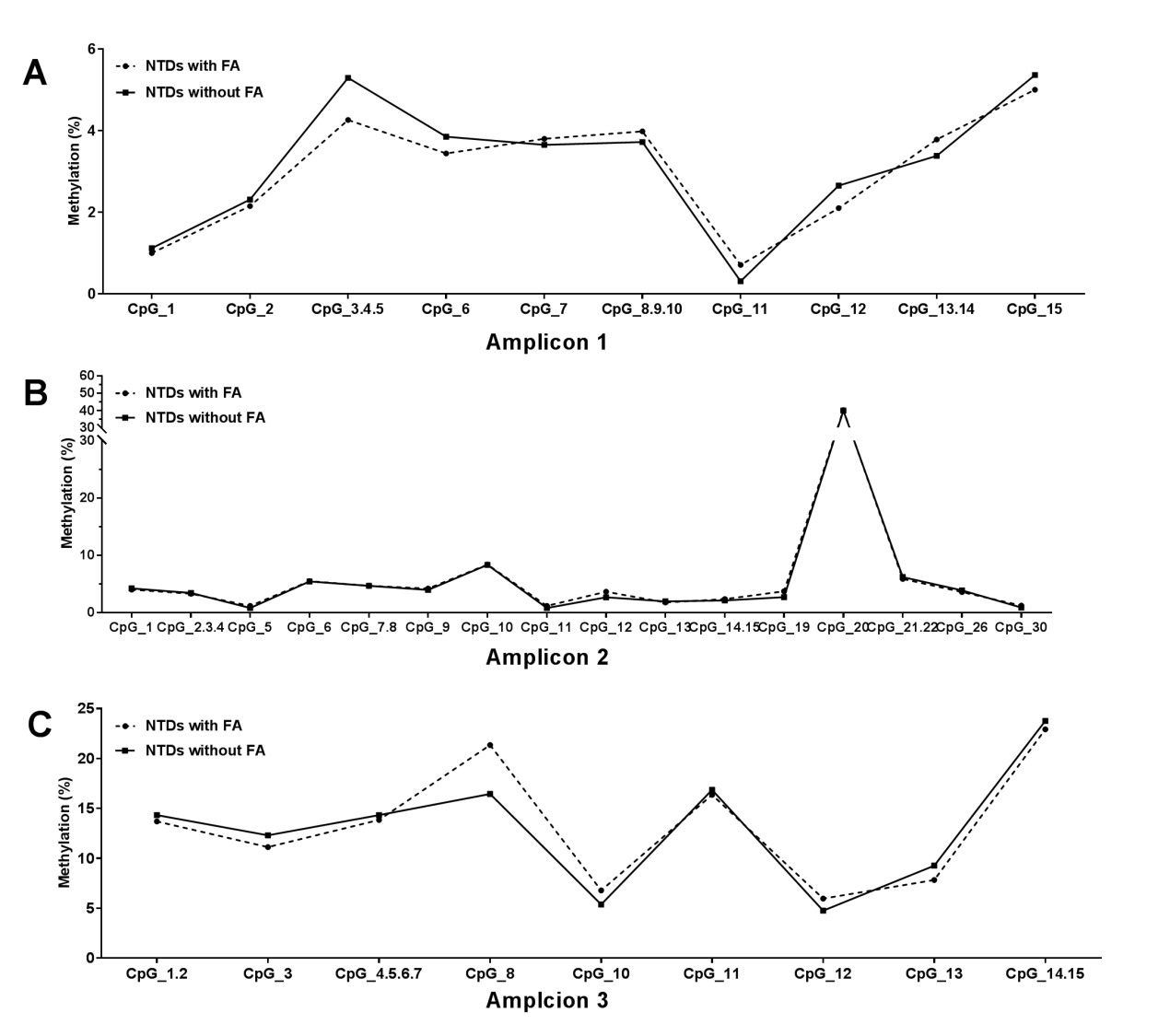


**Fig. S1** *PAX3* methylation pattern assayed by Sequenom EpiTYPER in NTD cases with and without folic acid supplementation. (A-C) Methylation level for each CpG site between NTD cases with and without folic acid supplementation in promoter (A-B) and body region (C). TSS1500, TSS200, 5’URT and the 1st exon were defined as the promoter region of *PAX3* gene in this study. The significance of differences was calculated using Independent t-test. FA, folic acid.
