# Supplementary material for: Aberrant methylation of Pax3 gene and neural tube defects in association with exposure to polycyclic aromatic hydrocarbons
Source: Clin Epigenetics. 2019 Jan 21;11:13. doi: 10.1186/s13148-019-0611-7 (PMC6341549; doi:10.1186/s13148-019-0611-7)
Supplement: Supplementary file 5 — Table S4. The PCR primer sequences in Sequenom EpiTYPER sequencing. (DOCX 15 kb) [file 13148_2019_611_MOESM5_ESM.docx]

**Table S4.** The PCR primer sequences in Sequenom EpiTYPER sequencing

| PCR products | Sequence |
| --- | --- |
| Amplicon 1F | aggaagagagAGTAATTGTTATTTTTTTGGGGTTG |
| Amplicon 1R | cagtaatacgactcactatagggagaaggctTTTTACCCAAAACTTAATCAAAAACC |
| Amplicon 2F | aggaagagagTTATTAGAGATGGGAAGAGAAAGTGG |
| Amplicon 2R | cagtaatacgactcactatagggagaaggctTAAAAAACCCCTCCCCAAAAC |
| Amplicon 3F | aggaagagagGGGTTATTTAAAAGTTTTTAGGGTTG |
| Amplicon 3R | cagtaatacgactcactatagggagaaggctCCCTCATAAATAAATTACCTATCATACAA |
