# Supplementary material for: Aberrant methylation of Pax3 gene and neural tube defects in association with exposure to polycyclic aromatic hydrocarbons
Source: Clin Epigenetics. 2019 Jan 21;11:13. doi: 10.1186/s13148-019-0611-7 (PMC6341549; doi:10.1186/s13148-019-0611-7)
Supplement: Supplementary file 7 — Table S6. Sequences of primers for real-time PCR in mouse study. (DOCX 15 kb) [file 13148_2019_611_MOESM7_ESM.docx]

**Table S6.** Sequences of primers for real-time PCR in mouse study

| Primer | Forward sequence | Reverse sequence |
| --- | --- | --- |
| *Pax3* | GGCTTTCGAGAGAACCCACT | AGGTCTCCGACAGCTGGTAT |
| *Gapdh* | CAGTGAGAAAGTCGGAGTCA | GACACCCATCACAAACATGG |
